# University of Pennsylvania, Veterinary Department: Commencement

**Published:** 1891-07

**Authors:** 


*University of Pennsylvania, Veterinary Department: Commencement.*—The annual commencement of the Veterinary Department of University of Pennsylvania, was held in the Academy of Music, Philadelphia, June 11th; the orations and valedictory were delivered by students of the departments of Arts and Law.

The following gentlemen received the degree of Doctor of Veterinary Medicine (V. M. D.).

| | |
|---|---|
| Bartholomew, J. Cleaver, | Philadelphia. |
| Bear, Benj. S. J., | Mt. Joy, Pa. |
| Bickel, Samuel D., | Philadelphia. |
| Bunting, Elwood B., | Burlington, N. J. |
| Conard, Milton E., | West Grove, Pa. |
| Conrow, Abraham E., | Moorestown, N. J. |
| Edwards, Warren, | Philadelphia. |
| Entriken, Harry D., | Kennett Square, Pa. |
| Jolly, Charles R., | Atlanta, Ga. |
| Records, John H., | Lewes, Del. |
| Senseman, B. Frank, | Mechanicsburg, Pa. |
| Tag, William, Ph. G., | Philadelpeia. |
| Wheeler, Arthur S., | New Orleans, La. |

J. B. Lippincott prize of $100, for the best general average in examination for the three years' course, was awarded to Arthur Seaver Wheeler, of New Orleans, La.

Dr. Simon J. J. Harger has been promoted from the position of Assistant Professor of Veterinary Anatomy and Zootechnics to the full professorship of Veterinary Anatomy and Zootechnics.

Leonard Pearson, B. S., V. M. D., who is at present pursuing special studies in the Royal Veterinary School in Berlin, has been elected Assistant Professor of the Theory and Practice of Veterinary Medicine.

Dr. Alexander Glass has been elected Lecturer on the Theory and Practice of Canine Medicine, and Dr. Edwin P. Muir has been elected Demonstrator of Materia Medica and Pharmacy.

